# Mental Toughness in Competitive Tennis: Relationships with Resilience and Stress

**DOI:** 10.3389/fpsyg.2016.00320

**Published:** 2016-03-15

**Authors:** Richard G. Cowden, Anna Meyer-Weitz, Kwaku Oppong Asante

**Affiliations:** ^1^Institute of Psychology and Wellbeing, North-West UniversityPotchefstroom, South Africa; ^2^Discipline of Psychology, University of KwaZulu-NatalDurban, South Africa

**Keywords:** mental toughness, resilience, stress, sport, competitive tennis

## Abstract

The present study investigated the relationships between mental toughness (MT), resilience, and stress among competitive South African tennis players. A total of 351 tennis players participating at various competitive standards completed the Sports Mental Toughness Questionnaire, the Resilience Scale for Adults, and a modified version of the Recovery-Stress Questionnaire for Athletes. The results indicated that total MT was positively associated with total resilience (*r* = 0.59), but negatively associated with total stress (*r* = -0.44). The resilience subscales of perception of self, perception of future, social competence, and social resources, but not family cohesion, significantly predicted total MT (*R*^2^ = 0.35). Both total resilience and total MT significantly predicted total stress (*R*^2^ = 0.21). Based on the findings, interrelations between MT and resilience are explored, implications outlined, and additional research is suggested to ascertain the contextual relevance and outcomes associated with each construct in sport.

## Introduction

Highly competitive sporting contexts emphasize the necessity to emerge as victor and accentuate the *winning is considered the only option* mentality. With success often bestowed solely upon winners or champions, the pursuit of sporting achievement has generated interest in determining the underlying characteristics of successful athletes (Hardy et al., 2014). Recent research attention has been directed toward mental toughness (MT), which has developed considerably from a historically heuristic basis (e.g., Loehr, 1986) and a time in which “virtually any desirable positive psychological characteristic associated with sporting success (had) been labeled as MT" (Jones et al., 2002, p. 206).

There is growing consensus about the central components of MT (Crust, 2008; Gucciardi et al., 2011) and research has contributed to refining the conceptualization, operationalization, and measurement of the construct. However, despite the advancement in understanding MT, a single, homogenous, and collectively agreed upon definition of MT remains absent (Tibbert et al., 2015). For instance, Clough et al. (2002) provide a dispositional or trait-like definition of MT, Jones et al. (2002) define the construct in terms of what MT enables athletes to achieve, and Coulter et al. (2010) assert MT as a process involving the interaction between a selection of psychobehavioral characteristics, the environment, and outcomes.

The definitional discrepancies expound the conceptual ambiguity surrounding MT, including whether it is a personality disposition (Horsburgh et al., 2009) or is amenable to interventions and developmental experiences (Parkes and Mallett, 2011; Mahoney et al., 2014). In addition, prior research has evidenced sport-specific differences in the type (Gucciardi et al., 2008) and degree (Gucciardi, 2009) of MT, culminating in the development of sport-specific MT inventories [e.g., Cricket Mental Toughness Inventory (CMTI); Gucciardi and Gordon, 2009]. Though extensive research involving single sports has been conducted (e.g., rugby, soccer, Australian Rules football, and cricket), competitive tennis has received comparably less MT research attention (Cowden et al., 2014).

The uniqueness of competitive tennis participation suggests MT in tennis may differ from other sports. Specifically, tennis requires athletes to frequently adapt to a variety of environmental conditions (e.g., weather) as well as playing surfaces (e.g., indoor hard court, clay). Contrasting other sports (e.g., soccer), tennis players are restricted from communicating, interacting, or seeking guidance from their coaching or support personnel during competition (Cowden et al., 2014). Also, the year-round competitive tennis season requires athletes to sustain physical and mental performance levels for protracted periods of time. Therefore, investigating MT in tennis (and other sport codes) may provide further indications of sport-specific types and manifestations of MT.

Collectively, the aforementioned issues have contributed to critical debates on the conceptual make-up and measurement of MT (e.g., Clough et al., 2012; Gucciardi et al., 2012; Perry et al., 2013). Notwithstanding the continued deliberations on the concept and definition of MT, for the purpose of this study, MT is defined as a collection of reasonably stable, advantageous characteristics that facilitate positive responses to the demands and pressures of sport participation (Sheard et al., 2009).

### Related Constructs: The Exemplar of Resilience

Conceptual disparities also appear to relate to the similarities between MT and other related constructs that may have been appropriated into the underpinning of MT. For instance, Clough et al. (2002) used hardiness (i.e., having a personal sense of control, resolute commitment, and perceiving challenges as opportunities; Kobasa et al., 1982) as a framework for conceptualizing MT. Clough et al. (2002) suggest that, although the constructs are both distinct and related, *confidence* (as a component of MT) is the distinguishing attribute between the two. Along similar lines, Cowden et al. (2014) found a positive association between MT and learned resourcefulness (i.e., a collection of learned abilities that regulate potentially detrimental cognitive-affective experiences to sustain or maintain functioning; Rosenbaum, 1990). The finding was attributed to the plausible convergence or similarities between the constructs (i.e., comprised of several constituents, superior control), but the authors noted the unexplained variance of MT (38%) may signify divergence between the constructs (i.e., commitment, achievement motivation).

Resilience is another construct that is noticeably similar to MT. Like MT, the definition of resilience remains a contentious area (Herrman et al., 2011). Conceptual differences also exist, such as whether resilience is an outcome (Mancini and Bonanno, 2009) or a process (Luthar and Cicchetti, 2000). However, Windle (2011, P. 163) proffers a recent definition of resilience as “the process of effectively negotiating, adapting to, or managing significant sources of stress or trauma. Assets and resources within the individual, their life and environment facilitate this capacity for adaptation and ‘bouncing back’ in the face of adversity”.

The notion of effectively overcoming and dealing with pressure, challenges, and stressors has been explicated in several descriptions of MT (e.g., Clough et al., 2002; Jones et al., 2002; Bull et al., 2005), which is arguably the strongest link between the two constructs. Galli and Vealey (2008) found that athletes make use of personal, social, and cultural factors to overcome various adversities in sport (e.g., periods of performance slumps). Fletcher and Sarkar (2012) identified several types of stressors that Olympic athletes successfully overcame through the use of psychological factors (e.g., positive personality, motivation, confidence, focus) that promote facilitative responses and positive sport performance outcomes. These factors resemble a number of characteristics associated with MT, including optimism (Nicholls et al., 2008), confidence or self-belief (Clough et al., 2002; Gucciardi and Gordon, 2009), concentration (Jones et al., 2007), and achievement motivation (Connaughton and Hanton, 2009).

Due, in part, to the apparent overlap between MT and resilience, there have been suggestions of the interrelatedness between the constructs. Loehr (1995) was among the first to posit *emotional resilience* as an aspect of MT and recent qualitative investigations have asserted resilience as a subcomponent of MT in sport (Gucciardi et al., 2008; Coulter et al., 2010), which have culminated in selected MT instruments specifying resilience as a subscale (e.g., CMTI; Gucciardi and Gordon, 2009).

Mental toughness and resilience, however, are dissimilar in several ways. There are assertions that resilience applies primarily to negative contexts, whereas MT also applies to positive circumstances (Gucciardi et al., 2008; Sheard, 2013). MT represents a set of personal attributes that influence the manner in which adversity, challenges, and goals are appraised and approached (Gucciardi et al., 2009b). Resilience, on the other hand, is associated with the possession of and/or the presence of protective (e.g., personal, familial, community; Kumpfer, 1999) and vulnerability factors that influence the risk-positive adaptation relationship (Punamaki et al., 2006). Thus, unlike MT, resilience seems to include a range of influential qualities outside of the self (e.g., perceived social support; Fletcher and Sarkar, 2012).

Perhaps for this reason, Gerber et al. (2013a) examined and supported the protective resource quality of MT among adolescents that were clustered according to resilient outcome categories (i.e., well-adjusted, maladjusted, deteriorated, and resilient groups). The students clustered into resilient and deteriorated groups did not differ on baseline levels of MT, but the resilient cluster displayed greater MT at the 10-month follow-up. In another study, Gerber et al. (2013b) found that MT moderated the stress-depressive symptom relationship, with lower levels of depression among adolescents high in MT, regardless of stress levels. However, neither study directly measured resilience using a validated instrument, which precludes a clear determination of the relationship between MT and resilience. Nonetheless, the findings emphasize MT as a resilience resource or protective factor that moderates the association between risk and adaption levels to facilitate positive outcomes (Gerber et al., 2013a,b).

With the obscurity that exists about the manner in which MT and resilience are related, quantitative MT-resilience investigations may contribute to delineating the similarities and differences between the two constructs. This includes whether resilience is a component of MT, whether MT is a protective factor in the resilience process, or whether the relevance of one construct to the other is dependent on particular situations or contexts. For instance, MT may contribute to resilient outcomes as a personal protective factor or resource. Selected types of resilience, on the other hand, may apply to MT based on a specific type of situation, such as personal resources applying primarily during competitive situational adversity (e.g., when behind in a match) and social resources required following a disappointing loss in order to rebound quickly.

### Mental Toughness and Resilience: Positive Adaptation to Stress Commonality

During exposure to internal and external demands to which consequences are ascribed, the concept of stress refers to disequilibrium between such demands (i.e., stressors) and the ability to successfully meet them (McGrath, 1970; Fletcher et al., 2006). Acknowledging the potentially deleterious effects of stress on an athlete’s mental and physical well-being (Crocker et al., 2015), MT and resilience are both comparably important for successfully dealing with stressors or avoiding the effect of stress as athletes pursue performance excellence (Gucciardi et al., 2008; Fletcher and Sarkar, 2012).

Although few quantitative studies involving resilience and stress in sport have been conducted, MT has received some attention in relation to stress. Mentally tougher individuals tend to report stressors as being less intense, perceive greater control over the stressors they are confronted with (Kaiseler et al., 2009), and report lower levels of depression despite perceptions of stress (Gerber et al., 2013b). Due to the conceptual overlap in the ability to bounce back from or overcome adversity and stress between MT and resilience, determining whether resilience and MT are, conjunctively, associated with lower levels of stress may provide additional insight into the similarities and distinctions between the constructs.

The purpose of the present study, therefore, was to explore the relationships between MT and resilience, MT and stress, and the role of resilience and MT in relation to stress. In particular, the following hypotheses were tested: (a) MT would be positively related to resilience, (b) MT would be predicted by the resilience resource domains (i.e., subscales), (c) MT would be negatively related to stress, and (d) MT and resilience would both be predictors of stress.

## Materials and Methods

### Participants

The sample included 185 male and 166 female competitive South African tennis players ranging from 18 to 84 years of age (*M* age = 28.91 years, *SD* = 13.87). The participants had played tennis for a minimum of 5 years (*M* age = 16.87 years, *SD* = 12.17) and had competed in a competitive tennis event within the last 2 weeks prior to participation in the study. The participants were purposively recruited through local and national South African tennis organizations, universities, and tournaments. The participants competed at international (*N* = 33), national (*N* = 78), university team/league (*N* = 156), local county (*N* = 23), and county club (*N* = 61) tournament levels.

### Materials

#### Mental Toughness

The Sports Mental Toughness Questionnaire (SMTQ; Sheard et al., 2009) was used to assess MT. The SMTQ is a 14-item Likert-type instrument and a multidimensional measure of MT along three subscales: confidence (e.g., “I have qualities that set me apart from other competitors”), constancy (e.g., “I get distracted easily and lose my concentration”), and control (e.g., “I worry about performing poorly”). CFA evidenced strong support for the hierarchical three-factor model, with a goodness-of-fit index (GFI) of 0.95 denoting good model fit (Sheard et al., 2009). The coefficients between the higher-order factor of total MT and second-order factors of confidence (*r* = 0.72), constancy (*r* = 0.71), and control (*r* = 0.66) were considered acceptable. Correlations between confidence and control, confidence and constancy, and constancy and control were 0.28, 0.31, and 0.31, respectively (Sheard et al., 2009). The authors also reported acceptable internal consistency estimates for global MT and each of the subscales (α = 0.72–0.81; Sheard et al., 2009).

Providing evidence for the divergent validity of the measure, correlations between the SMTQ and the subscales on the Life Orientation Test, Personal View Survey III-R, and the Positive and Negative Affect Schedule were moderate and ranged from 0.23 to 0.38, 0.14 to 0.33, and 0.12 to 0.49, respectively (Sheard et al., 2009). The discriminative power of the SMTQ has also been demonstrated based on statistically meaningful differences between athletes of dissimilar competitive levels, ages, and gender, with higher scores for more advanced competitive levels, for older athletes, and males (Sheard et al., 2009).

In the present study, Cronbach’s alpha was 0.74 for total MT, 0.64 for confidence, 0.52 for constancy, and 0.67 for control. Although confidence and control evidenced questionable internal consistency (George and Mallery, 2003), alpha is susceptible to underestimating internal consistency on scales with low item numbers (Briggs and Cheek, 1986). As a result, the subscales of confidence and control were retained for statistical computations. The poor internal consistency for constancy, however, resulted in the exclusion of the scale from subscale analyses.

#### Resilience

The Resilience Scale for Adults (RSA; Friborg et al., 2005) was used to measure resilience. The RSA has been validated on several occasions (e.g., Hjemdal et al., 2011; Capanna et al., 2015) and is a comprehensive measure of resilience protective factors. The 33-item inventory is rated on a 5-point semantic differential scale with opposing attributes at each end of scale for each item. The questionnaire encompasses six domains of resilience: social competence (e.g., “When I am with others: I easily laugh – I seldom laugh”), social resources (e.g., “I get support from: Friends/family members – No one”), family cohesion (e.g., “In difficult periods my family: Keeps a positive outlook on the future – Views the future as gloomy”), structured style (e.g., “I am good at: Organizing my time – Wasting my time”), personal strength/perception of self (e.g., “My abilities: I strongly believe in – I am uncertain about”), and personal strength/perception of future (e.g., “My future goals: I know how to accomplish – I am unsure how to accomplish”).

Friborg et al. (2005) reported the internal consistency values for each subscale as ranging from 0.66 to 78. Research has supported the convergent, criterion, and discriminant validity (Friborg et al., 2003; Friborg et al., 2005; Hjemdal et al., 2011; Capanna et al., 2015) of the measure. In this study, Cronbach’s alpha for total resilience (0.89), perception of self (0.70), social competence (0.77), family cohesion (0.81), and social resources (0.81) were acceptable to strong (Nunnally and Bernstein, 1994). Despite the internal consistency for perception of future (0.67) and structured style (0.48) being questionable and poor, respectively (George and Mallery, 2003), alpha may be underestimated on scales with low item numbers (Briggs and Cheek, 1986). As a result, perception of future was retained in subsequent subscale analyses, though the structured style subscale was not.

#### Stress

A modified version of the Recovery-Stress Questionnaire for Athletes (RESTQ; Kellmann and Kallus, 2001) was used to examine stress. Specifically, only the stress-related items were administered to the participants. Therefore, 40 of the original 76 items were retained for use in the current study. The items are rated on a 7-point Likert-type scale anchored at 0 (*never*) and 6 (*always*) and address the degree to which the participants experienced the item in the past 3 days/nights. The items comprise 10 dimensions of stress (four items for each factor), which permits the assessment of the stress experienced by athletes in a broad range of aspects inside and outside of sport.

Seven of the scales measure a general stress domain, comprising the subscales of general stress (e.g., “I was fed up with everything”), emotional stress (e.g., “I felt anxious or inhibited”), social stress (e.g., “I was annoyed by others”), conflicts/pressure (e.g., “I couldn’t switch my mind off”), fatigue (e.g., “I did not get enough sleep”), lack of energy (e.g., “I was unable to concentrate well”), and somatic complaints (e.g., “I felt physically bad”). Three subscales measure sport stress, which are labeled disturbed breaks (e.g., “I could not get rest during the breaks”), burnout/exhaustion (e.g., “I felt burned out by my sport”), and fitness/injury (e.g., “I felt vulnerable to injuries”).

The inventory has been validated using PCA (Kellmann and Kallus, 2001), a finding that has been cross-culturally verified (e.g., Gonzalez-Boto et al., 2008; Nederhof et al., 2008). Similar internal consistency estimates have been reported across studies (Kellmann and Kallus, 2001; Gonzalez-Boto et al., 2008; Nederhof et al., 2008). Kellmann and Kallus (2001) also found appropriate test-retest reliability estimates and support for the construct validity of each subscale. In this study, the relevant subscales were summated to obtain general and sport domains of stress as well as total stress. The internal consistency estimates were strong for total (α = 0.96), general (α = 0.93), and sport (α = 0.86) stress.

### Procedure

Following the attainment of relevant gatekeeper permission and Ethical Clearance from the University of KwaZulu-Natal’s Human and Social Sciences Research Ethics Committee, various tournament, university, and tennis organization directors were approached to obtain permission to access the participants. Based on athletes’ availability, the self-completed questionnaires were administered in groups of 5–10 players. The purpose of the study was explained to the participants, informed consent was outlined, and each participant completed an informed consent document prior to his or her participation. For each instrument, the athletes were prompted to consider and respond to the items according to the extent to which each item applied to them in relation to their involvement in *competitive tennis*. The inventories required approximately 20 min to complete.

### Data Analyses

The Statistical Package for Social Sciences (SPSS 23) was used to conduct all statistical tests. Prior to proceeding with statistical analyses, the hypothesis testing assumptions, including normality and homoscedasticity, were performed. The descriptive statistics and Pearson correlations (one-tailed) that were used to examine the relationships between the MT, resilience, and stress scales are reported in **Table 1**. Multiple linear regression analyses, using the stepwise forward selection method (alpha to enter = 0.05, alpha to exit = 0.10), are presented in **Table 2**. An alpha level of 0.05 was used for all statistical tests. For each variable, greater scores are associated with higher levels of the characteristic.

**Table 1:** Pearson correlations among all scales and subscales.

Variable	(1)	(2)	(3)	(4)	(5)	(6)	(7)	(8)	(9)	(10)	(11)	(12)
**(1) Total mental toughness**	-	0.77**	0.74**	0.59**	0.53**	0.41**	0.35**	0.42**	0.43**	-0.44**	-0.46**	-0.32**
(2) Confidence	-	-	0.27**	0.48**	0.47**	0.31**	0.32**	0.29**	0.33**	-0.24**	-0.27**	-0.12^*^
(3) Control	-	-	-	0.33**	0.30**	0.24**	0.20**	0.26**	0.22**	-0.45**	-0.46**	-0.35**
**(4) Total resilience**	-	-	-	-	0.72**	0.65**	0.67**	0.81**	0.80**	-0.36**	-0.36**	-0.26**
(5) Perception of self	-	-	-	-	-	0.47**	0.38**	0.45**	0.46**	-0.32**	-0.34**	-0.20**
(6) Perception of future	-	-	-	-	-	-	0.25**	0.42**	0.36**	-0.23**	-0.22**	-0.20**
(7) Social competence	-	-	-	-	-	-	-	0.39**	0.46**	-0.23**	-0.26**	-0.12^*^
(8) Family cohesion	-	-	-	-	-	-	-	-	0.69**	-0.27**	-0.28**	-0.21**
(9) Social resources	-	-	-	-	-	-	-	-	-	-0.26**	-0.26**	-0.21**
**(10) Total stress**	-	-	-	-	-	-	-	-	-	-	0.97**	0.85**
(11) General stress	-	-	-	-	-	-	-	-	-	-	-	0.71**
(12) Sport stress	-	-	-	-	-	-	-	-	-	-	-	-
*M*	41.35	18.15	10.37	133.72	24.29	16.42	23.02	24.40	30.31	93.27	65.71	27.56
*SD*	4.85	2.36	2.34	15.79	3.45	2.83	4.45	4.37	4.23	29.76	21.99	9.83

*^∗^*p* < 0.05 (one-tailed), ^∗∗^*p* < 0.001 (one-tailed)*.

**Table 2:** Multiple linear regression analyses (stepwise forward selection) predicting mental toughness and stress.

Model		DV = Total mental toughness			DV = Total stress		
	Predictor	*B*	β	95% CI for B	*B*	β	95% CI for B
Model 1	(Constant)	17.03		[13.45,20.61]			
	Perception of self	0.46^∗∗^	0.33	[0.32,0.61]			
	Social resources	0.20^∗∗^	0.18	[0.08,0.32]			
	Perception of future	0.26^∗∗^	0.15	[0.09,0.43]			
	Social competence	0.12^∗^	0.11	[0.01,0.23]			
	Family cohesion	-	-	-			
	*R*^2^	0.35					
	*F*	45.59^∗∗^					
							
Model 2	(Constant)				220.23		[193.51,246.95]
	Total mental toughness				-2.20^∗∗^	-0.36	[-2.91,-1.49]
	Total resilience				-0.27^∗^	-0.14	[-0.49,-0.05]
	*R*^2^				0.21		
	*F*				46.13^∗∗^		

*CI = confidence interval. ^∗^*p* < 0.05, ^∗∗^*p* < 0.001*.

## Results

### Relationships Among All Variables

Total MT was positively correlated with total resilience (*r* = 0.59) and the subscales of resilience (*r* = 0.35–0.53), which, according to Cohen’s (1992) effect size standards, were medium to large in effect size. Total MT correlated negatively with total stress (*r* = -0.44), general stress (*r* = -0.46), and sport stress (*r* = -0.32), which were medium and medium-to-large in effect size (Cohen, 1992).

### Predicting Mental Toughness: Resilience

Multiple linear regression was used to determine the extent to which the five subcomponents of resilience (the structured style subscale was excluded) predicted total MT. Perception of self (β = 0.33, *p* < 0.001), social resources (β = 0.17, *p* = 0.001), perception of future (β = 0.15, *p* = 0.003), and social competence (β = 0.11, *p* = 0.028) were statistically significant predictors of MT (*F* = 46.07, *R*^2^ = 0.35, 95% CI for *R*^2^ [0.27–0.43], *p* < 0.001; see **Table 2**). The family cohesion (*p* = 0.218) subscale was not a statistically significant predictor of total MT.

### Predicting Stress: Mental Toughness and Resilience

Multiple linear regression was computed with total stress as the dependent variable and total MT and total resilience as the predictors. The results indicated that total MT (β = -0.36, *p* < 0.001) and total resilience (β = -0.14, *p* = 0.016) were both significant predictors of total stress (*F* = 46.13, *R*^2^ = 0.21, 95% CI for *R*^2^ [0.13–0.29], *p* < 0.001; see **Table 2**).

## Discussion

The purpose of the present study was to explore the relationships between MT, resilience, and stress among competitive tennis players. Based on the results, the hypotheses that (a) MT and resilience would be positively related, (c) MT would be negatively associated with stress, and (d) both MT and resilience would significantly predict stress were all supported. The hypothesis that (b) MT would be significantly predicted by each of the resilience subdomains was partially supported, with one subscale (family cohesion) not found to be a significant predictor of MT.

### Mental Toughness and Resilience

The strong positive association between MT and resilience may demonstrate the conceptual parallels between the two constructs. That is, both resilience and MT are associated with effective adaptation, coping, maintaining functioning or performance, and achieving despite experiencing adversity, pressure, setbacks, or stress (Fourie and Potgieter, 2001; Clough et al., 2002; Galli and Vealey, 2008; Hosseini and Besharat, 2010). In fact, more recent resilience endeavors have outlined the core resilience protective factors among athletes, many of which are remarkably similar to the attributes of MT (e.g., confidence; Fletcher and Sarkar, 2012). This apparent resilience and MT overlap is further evidenced in the large amount of MT variability (i.e., 35%) that was explained by the resilience subscales of perception of self, perception of future, social resources, and social competence.

The finding that resilience resources did not account for approximately 65% of the variability in MT may support the contention of resilience as a component of MT (Coulter et al., 2010; Sheard, 2013). That is, if resilience is a characteristic of MT, it may be one of several MT subcomponents (Gucciardi et al., 2008; Gucciardi and Gordon, 2009). On the other hand, a range of social, familial, community, and cultural resilience protective factors have also been identified in non-sport (e.g., Cowen et al., 1997; Honey et al., 2011) and in athletic populations (e.g., Sarkar and Fletcher, 2014). Although external protective resources may be related to MT (e.g., social resources), or, perhaps contribute to MT development (e.g., supportive social environment; Mahoney et al., 2014), the internal, individual, and personal nature of MT suggests that such facets of resilience are unlikely to comprise MT (Chang et al., 2012; Hardy et al., 2014). This may be supported by the present finding that both MT and resilience, collectively, significantly predicted stress. Therefore, both MT and resilience appear to contribute to the variability in stress levels beyond that of either construct alone, a reflection of the divergent components that comprise MT (i.e., internal) and resilience resources (i.e., internal and external).

There may also be theoretical and conceptual distinctions between MT and resilience. For instance, Sarkar and Fletcher (2014) discern adversity (i.e., an incident with negative connotations) from stressors (i.e., environmental demands), with adversity only considered to have occurred if post-stressor maladaptive behavior is representative of that which may be experienced by a typical member of the population. For athletes, who are exposed to many types of stressors that members of the normative population may not experience, perhaps resilience only applies when they experience stressors that may subsequently be considered adversity for an average member of the population (e.g., loss of a close family member).

Mental toughness, on the other hand, may apply more specifically to stressors and the resultant stress that athletes experience during sport training, competition, and post-competition. Thus, although resilience may have a role in relation to overcoming stress, MT might be more important for negating or avoiding the detrimental effects of stress among athletes in sporting contexts, whereas resilience may be relevant primarily when experiencing more severe adversities that are likely to occur outside of sport participation.

### Mental Toughness and Stress

The finding that MT is related to lower levels of stress corresponds with the assertion that MT is associated with positively adapting to stress (e.g., Jones et al., 2002; Gucciardi et al., 2009a). The results support prior research that has reported mentally tougher athletes experience lower levels of stress or self-perceived stressor severity (e.g., Middleton et al., 2004; Horsburgh et al., 2009; Kaiseler et al., 2009), though it extends the MT-stress relationship to a sport-specific context, competitive tennis. As Kaiseler et al. (2009) suggest, mentally tough athletes might appraise stressors as less intense, resulting in a more optimistic outlook and a lower stress response. It may also relate to their superior ability to control their thoughts and emotions (Golby and Sheard, 2004; Crust, 2009) that assists with the appraisal of stressors or their ability to employ more effective coping strategies (Nicholls et al., 2008; Crust and Keegan, 2010). Alternatively, mentally tough individuals may use a range of psychological strategies (e.g., relaxation techniques, self-talk, mental imagery) that assist them when they encounter stressors or experiences stress (Crust and Azadi, 2010; Mattie and Munroe-Chandler, 2012).

### Future Research Suggestions

The findings in this study offer some key prospective directions for MT research. Specifically, a number of external resilience protective factors (e.g., social resources and social competence) were associated with greater MT, suggesting that a strong social support system, social interconnectedness, and interpersonal skills may be beneficial to MT. Similar types of external factors have been identified as important to the development of MT, including various interpersonal relationships (Bull et al., 2005; Connaughton et al., 2008, 2010; Gucciardi, 2011). Fostering an athlete’s social skills, a network of meaningful others (e.g., coaching team, peers), and a socially supportive environment, both in and outside of sport, may have positive implications for MT (Crust and Clough, 2011). Therefore, future research could determine the extent to which interventions aimed at building athletes’ social resources and interpersonal functioning, such as relationships with coaches and family, develop MT.

Based on the negative relationship between MT and stress found in this study, identifying sources of stress as well as an athlete’s psychophysiological reactions to such may enable the alteration of debilitative perceptions and responses. Initial evidence suggests that mentally tough athletes’ more opportunistic or facilitative appraisal of stressors assists them to experience lower stress responses (Kaiseler et al., 2009). Accordingly, investigating mentally tough athletes’ cognitive framing of different stressors appears to be an important area to pursue, along with determining the potential to develop MT through challenging and altering athletes’ dysfunctional appraisals of stressors (i.e., cognitive reframing; Crocker et al., 1988).

Another method through which negative stress responses may be avoided is through mentally tough athletes’ apparent attentional control abilities (Gucciardi and Gordon, 2009). With the propensity to sustain concentration levels, remain focused, and avoid distractions, athletes high in MT may be less inclined to devote attention toward certain types of stressors (e.g., adverse weather conditions), and, resultantly, experience lower levels of stress. Prospective research could assess the propensity for mentally tough athletes to attend to certain stimuli and not others. Similarly, experimental designs examining the cognitive distractibility profiles of athletes high and low in MT are encouraged, particularly in relation to stressors that vary in severity.

Future research is also necessary to determine mentally tough outcomes or indicators (e.g., behaviors) as a function of sport type and situations that require MT in sport. Research differentiating MT and resilience is also warranted, including distinctions along the lines of the relevance, application, and outcomes associated with each construct in varied sport and non-sport contexts.

### Limitations

There are selected limitations associated with the present study. Specifically, the cross-sectional design restricts conclusions of causality between the variables. Attempts to differentiate MT and resilience may require delineating the divergent circumstances in which the constructs may be relevant. Accompanying this is the necessity to generate consensus on the conceptualization of each construct as well as key definitions of the antecedents and consequences linked to both constructs (e.g., adversity, stressors, positive adaptation, outcomes). The sport specific focus of this study (i.e., tennis), as well as the inclusion of competitive tennis participants from South Africa, limits the ability to generalize the findings to other population groups. However, with sport-specific MT studies recommended (e.g., Crust, 2008) and indications of cross-cultural MT differences (e.g., Xinyi et al., 2004), this study afforded additional detail about the sport and culturally specific nature of MT.

In addition, although the RSA and SMTQ have been validated previously, the internal consistency of one subscale from each instrument was particularly low (<0.60), which limited the application of these subscales in this study. With prior studies raising internal consistency concerns over selected SMTQ subscales (c.f. Crust and Swann, 2011) and recent SMTQ conceptual breadth criticisms (c.f. Gucciardi et al., 2011), it is suggested that further use of the SMTQ follows refinement of the instrument. Also, as resilience in sport applications advance, it may be necessary to develop, refine, and validate a measure of resilience for specific use among athletes.

## Conclusion

The current study offers preliminary quantitative support for the interrelatedness between resilience and MT and extends the negative association between MT and stress to competitive tennis players, an athletic domain in which MT has been underexplored. The findings also offer initial evidence to suggest the distinct roles of resilience and MT in avoiding or alleviating stress, at least in competitive tennis. Additional research in this area is needed, especially along the lines of discerning MT from resilience and determining the contextual relevance of each construct in sport.

## Author Contributions

All authors listed, have made substantial, direct and intellectual contribution to the work, and approved it for publication.

## Conflict of Interest Statement

The authors declare that the research was conducted in the absence of any commercial or financial relationships that could be construed as a potential conflict of interest.
